# Comparison of bacterial isolated from burn wounds in ICU and burn ward patients

**DOI:** 10.1186/1753-6561-5-S6-P76

**Published:** 2011-06-29

**Authors:** V Mano, G Kasmi, B Byku, I Kasmi

**Affiliations:** 1 Laboratory of Microbiology, University Hospital Center "Mother Teresa", Tirana, Albania; 2 Pediatric, University Hospital Center "Mother Teresa", Tirana, Albania

## Introduction / objectives

Bacterial infection is of great importance in the course and prognose of burns. Thermal injury creates conditions for the growth of bacteria from the patients own body as well as environmental flora and also depresses host defence mechanisms.

We present the results of isolates from wounds in ICU and burn ward patients.

## Methods

We collected swabs from wounds in 16 ICU patients and 58 of burn ward patients during a two month period Jan.-March 2011. Clinical samples were inoculated on Blood agar and Mac- Conkey agar. The isolates were identified with Gram stain, oxydase test and Enterosystem 18R for the gram negatives and Staphy slide test for the staphylococci. The antibiogram was performed by the disc-diffusion method, (according to the NCCLS standards).

## Results

85% of the patients in the ICU carried Acinetobacter baumanii, 76% carried both Acinetobacter baumanii and Staphylococcus aureus, both of them very resistant strains. Two patiens carried even a third strain, that of Proteus mirabilis which was partly resistant to antibiotics. While the strains isolated from the burn ward consisted of 42,2 % Pseudomonas aeruginosa, 33,7% of S. aureus, 8% Acinetobacter baumanii. The remaining part consisted of Proteus mirabilis, Escherichia coli, Citrobacter freundi, Enterobacter sp.

Acinetobacter was resistant to: Gentamycin, Amikacin, Piperacillin, Ampicillin, Cefotaxim, Cefuroxim, and Ciprofloxacin. All Acinetobacter isolates were susceptible to Piperacillin + Tazobactam and Doripenem.

While S.aureus was susceptible to Eritromicin and Vancomicin and resistant to others.

## Conclusion

We found that Acinetobacter baumanii was the dominant strain in the ICU of burn ward and it has a positive relation to the length of hospitalization in ICU.

## Disclosure of interest

None declared.

